# Weight management recommendations for youth with Down syndrome: Expert recommendations

**DOI:** 10.3389/fped.2022.1064108

**Published:** 2023-02-03

**Authors:** Lauren T. Ptomey, Nicolas M. Oreskovic, James A. Hendrix, Dominica Nichols, Stamatis Agiovlasitis

**Affiliations:** ^1^ Department of Internal Medicine, University of Kansas Medical Center, Kansas City, KS, United States; ^2^ Departments of Internal Medicine and Pediatrics, Massachusetts General Hospital, Boston, MA, United States; ^3^ Department of Pediatrics, Harvard Medical School, Boston, MA, United States; ^4^ LuMind IDSC Foundation, Burlington, MA, United States; ^5^ Department of Kinesiology, Mississippi State University, Mississippi State, MS, United States

**Keywords:** weight loss, weight gain, obesity, dietary intake, exercise, physical activity, down syndrome, intellectual disability

## Abstract

Youth with Down syndrome (DS) have a higher prevalence of overweight and obesity compared to the general youth population. Due to physiological and cognitive differences observed in youth with DS, weight management recommendations developed for the general population, may not be suitable for youth with DS. However, there are no recent recommendations for weight management in youth with DS. A workgroup of clinicians and researchers with extensive experience working with youth with DS came together to give clinicians and families guidance for weight management in youth with DS. Recommendations were developed by the workgroup *via* a methodical, deliberative process. After the initial development of the recommendations, they were shared with an expert review panel and caregivers who rated the strength of the recommendation and strength of the evidence. The workgroup moved forward the recommendations which the review panels rated as strong. Eight recommendations were developed which focused on screening for overweight and obesity, screening for health conditions that may impact dietary intake and physical activity, screening for feeding difficulties, setting appropriate recommendations for dietary intake and physical activity, and well as prevention and treatment of overweight and obesity using evidence-based strategies. These recommendations can be implemented by clinicians working with youth with Down syndrome as well as the family, school, and other relevant entities.

## 1. Introduction

Obesity is one of the most prevalent chronic conditions, nearly one out of every five (19.3%) youth in the US are obese according to recent national statistics (1). Obesity disproportionately affects minority populations and people living in low-income communities (1). Being obese during youth increases the risk of being obese during adulthood (2) and has both short-and long-term health consequences (3). Children with obesity are at increased risk for: cardiovascular conditions including high blood pressure and high cholesterol, metabolic dysregulation including impaired glucose tolerance and Type 2 diabetes, breathing complications including asthma and sleep apnea, gastroesophageal reflux, gallstones, joint pains, fatty liver disease, low self-reported quality of life, low self-esteem, mental health problems including depression and anxiety, difficulties with academic performance, and social problems including stigma and being bullied (4, 5). Obesity can also increase the risk of complications for infections like influenza and COVID-19 (6). Beyond the individual, obesity has substantial societal effects by increasing healthcare costs – the combined annual direct and indirect costs of obesity in the United States totaled over $147 billion in 2008 (7). Finding ways to improve obesity treatment and self-management is therefore imperative.

Down syndrome (DS) is a genetic condition caused by extra chromosome 21 material in all or some cells of the body (8). The prevalence of DS among children aged 0–4 years in the United States is about 12.7 per 10,000 (9). Youth with DS have high risks for cardiac, metabolic, musculoskeletal, endocrine, respiratory, neurologic, and mental health conditions (10, 11). As well as a higher prevalence of overweight and obesity compared to the general youth population (12–16) which may further contribute to health risks (17). A 2016 review by Bertapelli et al. (18) reported that the combined prevalence of overweight [body mass index (BMI)-for-age 85th–94.9th percentile] and obesity (BMI-for-age ≥95th percentile) varied between studies from 23%–70%. A 2021 report in 122 youth with DS living in the United States, estimated the prevalence of overweight and obesity in youth with DS was 49% (14) compared to 39% in the general youth population (19). The onset of obesity in children with DS occurs around 2 years of age (12, 16). BMI rankings remain stable until puberty (∼12 years of age) when there appears to be an increase (12, 16). The etiology of obesity in youth with DS is unclear (20), but Bertapelli et al. (18) suggest the increased rates of obesity are associated with both physiological factors, such as increased leptin, decreased resting energy expenditure (REE), hypotonia, DS associated comorbidities, and lifestyle factors such as an unhealthy diet, and low physical activity levels.

Overweight and obesity in individuals with DS may contribute to health risks commonly observed in DS (21, 22). Controlling and monitoring weight status in youth with DS may reduce health risks during the growing years and possibly in adulthood. Weight management recommendations have been developed for the general pediatric population (23) as well youth with Autism (24); however, these recommendations may not be appropriate for adolescents with DS. Youth with DS have unique needs and challenges that make them vulnerable to risk factors in the obesogenic environment not shared by those in the general population by virtue of their limited cognitive abilities (25). Youth with DS have limitations with reasoning, money management, memory, and decision making, and require assistance from parents/guardians to complete activities of daily life (26). Additionally, parents/guardians of youth with DS report high levels of stress (27, 28), and often neglect their own health as they prioritize the needs of their children (29, 30). Additionally, individuals with DS have physiological profiles that may contribute to obesity and could impact weight loss (e.g., hypotonia, decreased REE, increased leptin, cardiac chronotropic incompetence). Thus, the daily life experience of adolescents with DS and their families is vastly different from their typically developing peers. Qualitative data indicate that clinicians face uncertainties when providing weight management for youth with DS, and many report lack of resources on the topic. In 2010, Murray and Krause published Recommendations for Obesity Management in Children with Down Syndrome (31); however, the evidence base on this topic has significantly increased in the decade following this publication. Additionally, these recommendations were developed specifically for clinicians, and do not provide families with any guidance. The goal of this paper is to equip clinicians and caregivers with updated accessible state-of-the-science recommendations for behavioral weight loss and the prevention of excess weight gain in youth with DS.

## 2. Development of weight management recommendations

A workgroup of clinicians and researchers with extensive experience working with youth and adults with DS came together in 2021 to develop recommendations that offer guidance to clinicians and families for behavioral weight management in youth with Down syndrome. This workgroup consisted of 1 Doctor of Medicine, 2 Registered Dietitians, one who is an expert in weight management, 1 non-profit foundation scientist, and 1 physical activity specialist, all with at least 10 years of experience working with individuals with DS. Two of the workgroup members were primarily clinicians, 2 were primarily researchers, and 1 had about equally split roles as clinician and researcher.

The following recommendations were developed *via* a methodical, deliberative process. Workgroup members participated in monthly conference calls between May 2021 and April 2022. They reviewed relevant extant research that focused on obesity in youth with DS, weight management interventions for youth with DS, co-occurring conditions in DS that could impact dietary intake and physical activity, and weight management guidelines for typically developing youth. Clinical consensus was achieved iteratively; the workgroup held extensive discussions focused on developing guidance for clinicians and families considering the lack of evidence-based weight management or weight loss approaches developed specifically for youth with DS. The workgroup consulted with other clinicians who provided either general medical care or weight management for individuals with DS to review standard of care, best practices, and clarify practices that deviated from published literature. For example, the workgroup consulted a special needs weight management clinic, a DS medical clinic, and authors of key peer-reviewed publications.

Using peer-reviewed literature, data collected from external clinicians, and their own experiences working with youth with DS, the group initially developed 9 recommendations. These recommendations were then shared with an expert review panel, who are members of the Down Syndrome Medical Interest Group (US-DSMIG). Each member of the expert panel was asked to read each recommendation and give feedback on both the strength of the recommendation and strength of the evidence. We also conducted a focus group with the expert panel in which they provided additional feedback on aspects of the recommendations to refine, clarify and, in some cases, to expand upon. Recommendations which did not demonstrate strength of the recommendation and strength of the evidence were removed (*n* = 1). The remaining 8 recommendations were updated based on feedback from the expert panel (e.g., providing additional detail or modifying the language). The remaining recommendations were shared with two panels of caregivers who provided feedback on the clarity of the recommendations. The caregivers were also asked to comment on how feasible it would be to implement the recommendations based on their lived experiences. The recommendations were again modified based feedback from caregiver panel and included adding additional details about physical activity.

## 3. Recommendations

### 3.1. Recommendation #1. Youth with down syndrome should be screened routinely for overweight and obesity

For youth with DS under the age of 2, clinicians should monitor weight and follow weight-for-length trends at each health care visit. The infant's growth should be plotted on the Down syndrome-specific charts (32, 33) for weight, length, weight for length, and head circumference and reviewed with the family (34).

For youth with DS 2 years of age or older, weight and height should respectively be measured on a standard stadiometer and scale, ideally with the individual in a gown without shoes on and plotted on the Down syndrome-specific charts for weight and height. Universal calculation and classification of body mass index (BMI), calculated as weight (kg)/height(m)^2^, is recommended for routine visits, and at least annually starting at the age of 2 years (23). BMI is correlated with more direct measures of body fat, and BMI classification serves as the first step in assessment of obesity (23). Weight status classifications are based on BMI. For children in the United States, sex-specific BMI-for-age percentiles are calculated relative to the 2000 US Centers for Disease Control and Prevention (CDC) growth reference (35). The BMI can then be classified as underweight (<5th percentile), healthy weight (5th to <85th percentile), overweight (85th to <95th percentile), and obesity (≥95th percentile). Severe obesity is defined as a BMI ≥120% of the age- and sex-specific 95th percentile or an absolute BMI ≥35 kg/m^2^, whichever is lower (25, 36). Additionally, specialty growth charts are available for youth with DS (32); however, while the DS specific growth charts are useful in comparing youth with DS to their DS peers, they do not appear to provide better classification of weight status or health risk for youth with DS over the age of 10 compared to the standard CDC growth chart (33, 34). Thus, as recommended by the 2022 Health Supervision Guidelines for Children and Adolescents with Down Syndrome, BMI should be plotted on the DS-specific growth chart for youth up to age 10, and for children over the age of 10, BMI should be plotted on both the DS-specific growth chart and the CDC growth chart (34).

Given that youth with DS have altered body composition including a higher prevalence of central adiposity compared to youth without DS (37) and that the extent to which BMI captures excess body adiposity in youth with DS is not known (33), the use of BMI alone may not be accurate enough to detect excess adiposity in this population. Thus, waist circumference should be measured routinely and at least annually. Waist circumference should be measured at the midpoint between the lower margin of the least palpable rib and the top of the iliac crest, using a stretch-resistant tape that provides a constant 100 g tension (38). The waist circumference to height ratio should also be calculated – a weight circumference to height ratio of >0.5 indicates excess central adiposity and is associated with higher risk of metabolic syndrome (37, 39).

### 3.2. Recommendation #2: clinicians and families should be aware of health conditions and risk factors that are common in down syndrome and may impact the development of obesity

DS is associated with several health conditions that have independent associations with dietary intake and physical activity pattern of youth with DS (40), and may contribute to the development of obesity (18). Clinicians working with youth with DS should screen for and monitor these health conditions to aid in the prevention or treatment of obesity, and families should be aware of how these risk factors may influence diet or physical activity. Table 1 lists medical conditions associated with DS and the condition's potential impact on weight management.

**Table 1:** Health conditions and risk factors common in youth with Down syndrome that can impact weight control *via* either diet or physical activity.

Medical condition	Impact on weight management	Impact on dietary intake	Impact on physical activity
**Sensory Input**			
Visual Impairment	Glaucoma, visual field defects, and keratoconus are diseases of the eye that can limit vision. Limited vision can impact feeding and participation in exercise and sports.	☑	☑
**Oral and Digestive System**			
Dental Disease	Certain dental conditions including delayed tooth eruption and missing teeth can make eating healthy foods more difficult.	☑	
Esophageal and Swallowing disorders	Esophageal disorders causing strictures along with other esophageal motility disorders can lead to food avoidance and result in decreased eating due to inability to properly swallow foods.	☑	
Intestinal obstruction, Hirschsprung Disease, Congenital Duodenal Atresia	Intestinal disorders characterized by obstruction can cause loss of appetite. Children with a prior surgical repair of the intestinal tract, such as a repaired congenital duodenal atresia, can develop a late-term complication called intestinal strictures, which can decrease intestinal motility and may consequently result in food avoidance.	☑	
Celiac Disease	Poorly controlled Celiac disease can cause inflammation of the cells lining the intestinal tract, resulting in poor absorption of nutrients and inadequate calorie absorption. Poorly controlled Celiac disease can also cause changes in bowel habits including increased stooling frequency and diarrhea, which can cause weight loss. Slight weight increases along with decreased appetite gain can also be the result of abdominal bloating, another symptom of poorly controlled Celiac disease. Following a gluten-free diet may result in low intake of fiber, Vitamin D, Vitamin B12, and folate. Many gluten-free food alternatives (e.g., gluten free cookies) are high in saturated fat, added sugar, and calories, promoting weight gain.	☑	
**Endocrine**			
Thyroid Disease	Inadequately controlled hypothyroidism can result in changes in appetite and weight changes. An under-functioning thyroid can cause fatigue and a decreased desire to engage in physical activity.	☑	☑
**Cardiovascular**			
Congenital heart disease	Uncorrected congenital heart disease can lead to decreased physical activity and participation in sports due to fatigue with exertion. Active congenital heart disease can lead to fluid retention presenting as weight gain.		☑
Acquired heart disease	Diseases of the heart valves can develop during childhood and adolescence that may cause fatigue and a decreased desire to engage in physical activity. Heart valve disease can cause fluid retention presenting as weight gain.		☑
Moya-Moya Disease	Strokes associated with Moya-Moya disease can limit physical activity.		☑
Vascular ring	Aberrant right subclavian artery can sometimes cause compression of the esophagus, limiting solid food intake.	☑	
**Pulmonary**			
Pneumonia and Bronchitis	Acute lung infections can cause systemic symptoms, including loss of appetite and when severe can result in decreased physical activity capacity.	☑	☑
Obstructive Sleep Apnea	Inadequately controlled sleep apnea can result in daytime fatigue and a decreased desire to engage in physical activity. Inadequately controlled sleep apnea may result in a lower metabolism, which can lead to weight gain. Use of CPAP may also lead to slight weight gain by decreasing your basal metabolic rate.		☑
**Musculoskeletal**			
Hypotonia (Low Muscle Tone)	Poor muscle tone can make participating in exercise and sports difficult, resulting in less physical activity. Low muscle tone can result in a decreased metabolism with lower daily energy requirements.	☑	☑
Arthritis (Joint Disease)	Arthritis and joint pain can limit physical activity. Some classes of medications used to treat inflammatory type arthritides, including steroids, can cause increased appetite as well as weight gain.		☑
Joint stability and Posture	Loose ligaments (ligament laxity) and other causes of poor joint stability can limit physical activity capacity. Poor or misaligned posture from flat feet and other common foot, ankle, and hip conditions can cause pain that limits physical activity.		☑
Osteoporosis	Low bone density is common in individuals with DS. Increased consumption of foods rich in calcium and vitamin D, as well as regular weight bearing physical activity can increase bone density and decrease the risk for bone fractures in individuals with DS.	☑	☑
Atlanto-axial instability (AAI)	Some sports and activities are contraindicated in individuals with atlanto-axial instability/subluxation.		☑
**Neurologic**			
Seizure disorder	Certain medications used to treat seizures can cause either loss of appetite or weight gain.	☑	
Regression disorder in DS	A decrease in global activity results in decreased eating and decreased physical activity.	☑	☑
Developmental Delay	Autism Spectrum Disorder can impact eating and physical activity levels.	☑	☑
**Psychiatric**			
Mental Health (Anxiety, Obsessive Compulsive Disease, Depression, Schizophrenia)	Emotional eating can be seen with Anxiety and Depression which is often associated with increased caloric intake and consumption of unhealthy foods. Depression can cause a decreased desire to engage in physical activity. Antipsychotic medications can cause increased appetite and weight gain.	☑	☑
**Cancer**			
Leukemia, Testicular cancer	Cancer treatments can cause appetite loss and weakness resulting in decreased physical activity levels. Steroid medications can lead to increased appetite and weight gain.	☑	☑

### 3.3. Recommendation #3: clinicians should screen for feeding difficulties in all youth with down syndrome

Feeding difficulties are common among infants, children, and adolescents with DS (41). Feeding difficulties, changes in feeding, or changes in respiratory symptoms with feeding should be reviewed during medical visits (34).

Early feeding difficulties can result in protein-calorie malnutrition or inadequate fluid intake, and may require increased calories, modified textures, modified consistency, or alternative methods of feeding to achieve adequate weight gain with minimal aspiration risk (41–43). Older children with DS may continue to have difficulty with hard textures, foods with multiple consistencies or those that require more chewing or sensory tolerance like raw vegetables, unpeeled fruit or less-processed cooked meats or fish (43).

Maladaptive mealtime behaviors can make weight management harder. Caregivers of youth with DS report high frequency of food selectivity, continued eating in the presence of food, swallowing without enough chewing, and eating (or drinking) large amounts of food (or caloric beverages) in short periods of time (43–45). Eating large amounts of food in a short period of time is a predictor of rapid weight gain and higher body fat in preschool-age children (46). Youth with DS should have early and continued access to support for developing and maintaining skills in functional chewing, food preparation, and self-feeding, with focus on less processed alternatives to ultra-processed foods.

### 3.4. Recommendation #4: clinicians should include assessments of dietary intake and physical activity at every visit

Collection of dietary intake and physical activity are essential for prescribing appropriate energy intake for both weight loss and maintenance, providing feedback to participants in weight management programs, describing changes over time, and determining the effectiveness of the program.

A typical approach is to obtain a dietary intake is to have a parent or caregiver record all foods and beverages consumed for the 3-days prior to the medical visit using a simple diet journal or a smart phone app such as *Lose it!* or *MyFitnessPal*. Clinicians can then review the food record to estimate the patients average daily caloric intake, macronutrient intake, and the quality of the diet (for example daily servings of fruits and vegetables). This information should be used to create individualized diet goals; for example, increasing fruit and vegetables intake from 3 to 4 servings per day, reducing the number of sugar sweetened beverages (juice, soda) consumed from 3 to 1 per day, reducing the consumption of fast food or highly processed foods to 1 time per week, or reducing overall energy (calorie) intake. Additionally, the dietary record can be used to identify food aversions and food selectivity, such as rejecting certain foods based on color or consuming only certain textures, which can lead to nutritional deficiencies (47).

Assessment of physical activity can be done with self-reported questionnaires when a patient comes into the clinic or having the youth with DS wear a physical activity tracker. There are pros and cons to each method. While the use of a questionnaires is easy, can be done in clinic, and is inexpensive, the accuracy of these questionaries in youth with DS is unknown. Youth are less likely to make accurate self-report assessments due to developmental differences, especially in the ability to perform detailed recall and understand concepts regarding physical activity duration and intensity (48). This may be amplified in youth with DS who may have difficulties with cognitive functioning, memory, and attention. Recently, the American Academy of Pediatrics opted to not recommend a specific physical activity assessment tool because of limitations to existing questionnaires (49).

Conversely, physical activity trackers have grown in popularity; these include simple pedometers as well as wrist-worn physical activity trackers. Pedometers provide step-counts as a quantity of physical activity and can often be purchased at a low-cost; however, pedometers do not provide information regarding time or intensity of physical activity, and it may be difficult to obtain long-term data as most pedometers can only store 1–7 days of activity. Recently, wrist-worn physical activity trackers have become popular. These devices often combine an accelerometer to measure minutes of physical activity and steps as well as a heart rate monitor to measure physical activity intensity. These devices often sync with an app on a smart phone so the user can see physical activity data, including daily minutes of physical activity, steps, resting heart rate, and heart rate during exercise. These data can then be shared with the medical team of youth with DS. However, these trackers can be costly, and the heart rate and intensity data provided by the devices may not be accurate as youth with DS have chronotropic incompetence (50) and increased heart rate variability (51).

The physical activity data obtained by either questionaries or devices should than be used to determine level of physical activity, set goals for increased activity, review barriers to physical activity for families, and provide feedback regarding change in physical activity since the last visit.

### 3.5. Recommendation #5. Clinicians and families should set appropriate recommendations for dietary intake

Determining nutrition and energy (calorie) requirements is challenging because these vary depending on the severity of intellectual disability, mobility status, age, medications, and feeding problems (52). When determining energy requirements, it is important to individualize the requirements based on all these considerations, as well as monitor the individual and make changes to the plan of care as needed. Predictive equations, that rely on the individual's height and/or weight, are often used to determine the energy needs of an individual. However, youth with DS may have a significantly lower resting metabolic rate and total daily energy expenditure relative to body size and composition (53, 54); thus, many predictive equations overestimate the energy requirements for adolescents with DS. Recent literature (55) demonstrates that the estimated energy requirement equations developed for children by the National Academy of Medicine, previously the Institute of Medicine (IOM) (56) provides the most accurate prediction of energy needs in youth with DS. The IOM equations include both an equation for general use (i.e., applied only to healthy weight participants) or overweight/obese specific equation (i.e., used for participants who were overweight or obese). Table 2 provides the difference predictive equations by sex and weight status.

**Table 2:** The Institutes of Medicine estimated energy requirement equations.

Stratification	Predictive Equation
Healthy weight male	79 − 34.2 × age + 730 × height + 15.3 × weight
Healthy weight female	322 − 26 × age + 504 × height + 11.6 × weight
Overweight/obese male	420 − 33.5 × age + 418.9 × height + 16.7 × weight
Overweight/obese female	516 − 26.8 × age + 347 × height + 12.4 × weight

Once resting energy expenditure calculated using the IOM equations are calculated, it needs to be multiplied by an activity factor to yield total daily energy needs. The clinician should choose the best activity factor for the youth with DS based on the physical activity assessment collected during screening.

• For girls, use 1.1 for sedentary/non-active; 1.3 for active (3 or more days of vigorous activity of at least 20 min/day, OR 5 or more days of moderate-intensity activity or walking or at least 30 min/day); and 1.5 for very active (5 or more days of vigorous activity of at least 30 min/day, OR 5 or more days of moderate-intensity activity or walking or at least 60 min/day).
• For boys, use 1.1 for sedentary/non-active; 1.25 for active; and 1.4 for very active.

This will yield the approximate daily calories needs for weight maintenance. If weight loss is a goal, 250–500 calories should be subtracted from the total to get approximate needs for weight loss.

Currently, there is no evidence demonstrating that the macronutrient (i.e., fat, protein, carbohydrates) needs of youth with DS are different than youth without DS. Thus, it is recommended to refer to the national dietary recommendations when providing dietary recommendations for youth with DS. The 2020–2025 Dietary Guidelines for Americans were published in December 2020 (57). These Guidelines expand previous versions that provided a roadmap for chronic disease prevention through adequate nutrition and address the role of the food environment, including access to ultra-processed foods and the ease of preparing and consuming them. Furthermore, the 2020–2025 Dietary Guidelines now stress the importance of maintaining healthy dietary patterns across the lifecycle. According to the 2020–2025 Dietary Guidelines, starting at age 2 years, youth should limit added sugars to less than 10% of calories and saturated fat to less than 10% of total daily calories. Added sugar and added fat tend to be sources of “empty calories,” leaving less space for more nutrient-dense foods. Additionally, calories from sugary drinks make it difficult to maintain a healthy meal pattern and weight (58). Younger children should avoid foods and drinks with added sugars. Water should be offered (not juice or juice drinks) for the whole family with introduction at 6 months or as directed by a medical team. There is not one menu to solve the challenge of healthy eating. A diversity of nutrient-dense ingredients and foods can support a healthy eating pattern. This is true for all youth including those with DS even with personal preferences and cultural traditions considered.

### 3.6. Recommendation #6. Clinicians and families should set appropriate recommendations for physical activity

When developing a physical activity program as a component weight control in youth with DS, health professionals should consider current recommendations. The U.S. Department of Health and Human Services launched the second edition of the Physical Activity Guidelines for Americans in 2018, affirming that physical activity improves a wide range of health outcomes including but not limited to weight status and body composition in youth aged 3–17 years (59). The guidelines are generally the same for youth with and without disabilities. Children aged 3–5 years should perform a variety of physical activities throughout the day and should engage in active play such sports and interactive activities involving running, jumping, climbing, and crawling, among others. It is also recommended that youth aged 6–17 years obtain ≥60 min of moderate-to-vigorous physical activity daily accumulated in bouts of different durations and should include vigorous physical activity, muscle-strengthening activity, and bone strengthening activity at least 3 days/week. The guidelines call for physical activities that are age-appropriate, enjoyable, and offer variety.

Most youth with DS across the lifespan do not meet recommendations for physical activity and have high levels of sedentary behavior (60–62). While youth with DS should be encouraged to meet the recommendations, this may not be immediately attainable for some youth with DS. Thus, physical activity programs must have gradual progression in frequency, duration, and total weekly amount of physical activity (59). Table 3 provides an example of a gradual exercise progression plan for youth aged 6–17 years which has been previously implemented in youth with DS (63). Most of the proposed amount of physical activity shown should involve moderate aerobic activity (e.g., brisk walking, hiking, and bicycle riding), but also include three times per week (a) vigorous aerobic activities (e.g., running and sports), (b) muscle-strengthening activities (e.g., resistance exercises using body weight), and (c) bone-strengthening activities (e.g., hopping, skipping, and jumping rope). Importantly, physical activities should be enjoyable. Enjoyment during physical activity may be achieved by affording youth with opportunities for engaging in preferred activities (e.g., if someone enjoys music, have dance parties with favorite songs), acquiring movement skills, interacting with others, and gaining positive feelings through movement, while avoiding pain, frustration, and an emphasis on competition (64, 65).

**Table 3:** Example of an exercise progression plan for youth with Down syndrome 6–17 years of age.

Week	Days/week	Min/day	Min/week
1	3	15	45
2	3	20	60
3	4	20	80
4	4	25	100
5	4	30	120
6	5	25	125
7	5	30	150
8	6	30	180
9	6	35	210
10	6	40	240
11	7	40	280
12	7	45	315
13	7	50	350
14	7	60	420

Furthermore, parents and clinicians should be aware that the minimum recommended amount of physical activity is intended for attaining general health benefits, but they are not specific for weight loss in youth with obesity. However, physical activity is associated with increased cardiovascular fitness (66), muscular strength and endurance (67), and reduced risk of chronic disease (68) in youth with DS. These benefits are independent of its impact on weight, and any increases in physical activity or decrease in sedentary behaviors, such as screen time, may have health benefits (69, 70).

Prior to recommending physical activity programs, especially of vigorous intensities, health professionals should conduct an evaluation of current physical activity levels with signs and symptoms of cardiovascular, metabolic, and other health conditions. Based on this preparticipation screening, medical clearance may be required prior to starting the exercise program. It is also important for clinicians and families to address the barriers to physical activity youth with DS face and consider their physical activity preferences. Barriers within the person include acute health problems, low physical fitness levels, low motor skills, orthopedic anomalies, lack of energy, and boredom (60, 71, 72). Parental barriers include family structure (marital status, other siblings, etc.), lack of self-efficacy for encouraging activity in their adolescent, time constraints, lack of affordable/accessible transportation, and low rates of parental physical activity (73, 74). Environmental barriers include lack of accessible, inclusive, and adapted programs, limited assistance by professionals, negative attitudes towards people with DS, and limited friendships (60, 71, 72). Physical activity in youth with DS may be facilitated by health care professionals knowledgeable in designing programs, family members who understand their roles in modeling physical activity, and programs that are accessible, structured, adapted to the needs and abilities of youth with DS, and ones that promote social interactions and enjoyment (71, 73). Families should ensure their children wear well-fitting shoes with proper arch support to avoid complications of foot anomalies. Additionally, youth with DS, family members, and clinicians should be educated to recognize warnings of cardiac distress, such as palpitations, syncope, lightheadedness, and dyspnea. Commonly performed activities among individuals with DS are walking, dancing, swimming, bowling, and team sports, but there is variation in physical activity types around the world (60, 71, 72). Overcoming barriers and identifying facilitators and preferences of physical activity for youth with DS may increase the success of physical activity programs for weight control.

### 3.7. Recommendation #7. Clinicians should provide multi-component behavioral weight management treatment programs specific to the needs of youth with down syndrome and with overweight or obesity

Behavioral weight Management strategies for youth with DS are based on those for the general youth population. However, clinicians working with youth with DS must consider their specific needs and the needs of their families. The quality and quantity of data to base recommendations for effective weight management specifically for youth with DS is limited (25, 75–77). Most behavioral weight management interventions have been conducted in youth with intellectual and developmental disabilities and were comprised of physical activity alone (75–80), a combination of diet and physical activity (81), and multi-component interventions which included diet, physical activity, and behavioral/education strategies (25, 63, 82–88). This literature suggests that the most effective behavioral weight management interventions are multi-component interventions that include changes to diet and physical activity and behavioral strategies such as self-monitoring of diet and physical activity, mindfulness, and goal setting. Clinicians should provide guidance and direct families towards multi-disciplinary treatment programs when weight management is required to maintain health goals. It is important that this guidance be empathetic, inclusive, and honest to empower families to make informed decisions. The following are specific recommendations that should be considered when providing multi-component treatment programs for youth with DS.

#### 3.7.1. Contact hours

The US Preventive Services Task Force Recommendation Statement on Screening for Obesity in Children and Adolescents recommends ≥26 contact hours over 12 months to improve weight status in typically developing children and adolescents (89). However, the cost and scalability of high contact programs may be limiting factors in the feasibility of this approach. A study in adolescents with intellectual disabilities (*n* = 110, 48% with DS, age 13–21 yrs) that included 30–45-minute individual sessions with participants and parents twice per month across 12 months, a total of ∼18 h across 12 months which is less than the ≥26 contact hours over 12 months, still resulted in weight loss (63). This suggests that intervention programs with fewer contact hours may still be effective for weight loss in youth with DS.

#### 3.7.2. Delivery format

Limited evidence in typically developing children and adolescents with overweight/obesity suggests that weight loss achieved with tele-health behavioral interventions may be minimal (90) and that a combination of face-to-face and remotely delivered sessions may be required to elicit clinically relevant weight loss (91, 92). However, recent research in adolescents with intellectual and developmental disabilities including DS demonstrates that weight management can be successfully delivered in-person or by tele-health (45, 63, 74, 85). In a 2021 study, adolescents attended behavioral lifestyle sessions with a trained health educator every 2 weeks across a 6 month period (63). Participants enrolled in the in-person delivery arm met with a health educator during individual home-visits and self-monitoring of diet and physical activity was completed by participants using pencil and paper records. Participants in the tele-health arms met with a health educator remotely using FaceTime™ video conferencing and self-monitoring was completed using a web-based app for diet (Lose it!) an activity tracker for physical activity (Fitbit®). Results of the study found no differences in weight loss between the in-person or tele-health formats, suggesting that remote delivery is just as effective as face-to-face delivery. Additionally, tele-health may be useful in this population as it eliminates the need for parents to provide transportation to the intervention site and eliminates the time associated with travel.

#### 3.7.3. Family-Based

Multiple studies in youth with DS involved a designated study helper who worked with the participant and engaged in the intervention themselves through attendance to meetings or sessions (25, 81–83, 85). Other studies were family-based trials (25, 82, 84, 87), which included significant parental involvement in family exercise, nutritional education sessions, as well as behavioral or motivational education sessions to assist with making healthier lifestyle changes at home. One study (25) compared an intensive 16-week educational approach that included nutrition and physical activity education with the same approach, but with increased parental support and training in 21 adolescents with DS. The rigorous parent training intervention guided parents to work with their adolescent son or daughter with DS to track diet and physical activity, set specific weekly goals for both, participate in a weekly phone call from the interventionist to track progress on both, report and discuss results with fellow parents and a behavioral therapist weekly, and receive feedback and reinforcement. Results of that study indicated that significant involvement of family members yields greater weight loss. A subsequent trial (86) examined the long term changes in weight after use of the increased parental support and training intervention in a sample of youth with intellectual disabilities (*n* = 24, 56% DS, age 14–22 yrs.) and included three 45-minute in-person group sessions per month (2–5 participants and parent) and one monthly 45-minute session with participants and parents separately during weight loss and 2 sessions per month during weight maintenance which alternated between 90-minute in-person group sessions (participants and parent) and 30-minute individual parent sessions, in a group setting. Participants who followed the intensive family-based program for 12 months had a mean weight loss of 6.1 kg, 6 months after ending the intervention, suggesting that the family-based approach is successful for both weight loss and long-term weight maintenance.

Research findings also indicate that family members significantly influence the physical activity levels of their children with DS. A recent cross-sectional review examined different intrapersonal, interpersonal, and environmental factors associated with device-measured physical activity in 92 adolescents with intellectual disabilities (55% DS, 11–21 years) and determined that the factor that most influenced physical activity levels of adolescents with intellectual disabilities was parental physical activity (93). Results suggest that for every 10 min of moderate to vigorous physical activity the parent performed, adolescents with intellectual disabilities achieved 6 min of moderate to vigorous physical activity. Notably, the correlation between parental physical activity and adolescent physical activity was higher in adolescents with DS compared to those with other intellectual disabilities. It should be noted that parents of youth with DS face emotional and physical challenges that exceed those of parents caring for typically developing adolescents. Thus, clinicians should assess family stressors and consider the family environment before making recommendations for family-based weight management treatments.

#### 3.7.4. Dietary components

Nearly all the evidence for effective weight management interventions in youth with DS includes components that focus on healthy eating and physical activity. The specific dietary recommendations vary widely among studies, so no one unified approach has demonstrated superiority over another. Common components of successful diet interventions in youth with DS are: providing specific recommendation for the number of servings from each food group; encouraging foods with low energy density (e.g., fruits, vegetables, and lean meats) and limiting foods with high energy density (e.g., sweet and fried foods); avoiding dietary restriction; allowing individuals to have their favorite foods in moderation; and tracking dietary intake using simple pictorial displays (25, 52, 63, 84, 86). Additionally, portion-controlled meals have been shown to be effective for weight loss in youth with DS (63, 85). For example, portioned lunch boxes or single-serve containers that are a “just right” size for the individual can help families and caregivers support the routine of adequate nutrition at home. A dietitian can provide individualized assessment and provide guidance specific to any child or adolescent. Specific recommendations for portion sizes may vary from individual to individual based on physical activity level, age, gender, weight status, and height (as discussed earlier). This is one reason that healthy eating and physical activity promotion are effective partners in weight management. Table 4 provides some online resources that can help with the promotion of healthy eating specific to those with DS.

**Table 4:** Technology based tools for self-monitoring and online programs for the promotion of health eating and physical activity.

DIET	PHYSICAL ACTIVITY
***Tools for Self-Monitoring of Diet***	***Tools for Self-Monitoring of Physical Activity***
MyFitnessPal (app)	Fitbit Tracker
Lose it (app)	Garmin Vivofit Tracker
	Apple Watch
	Any Smart Phone
***Online Tools for Promotion of Healthy Eating***	***Online Tools for Promotion of Physical Activity***
The Accessible Chef: https://accessiblechef.com/	Special Olympics Fit 5: https://resources.specialolympics.org/health/fitness/fit-5
Gigi's Kitchen Online: https://gigisplayhouse.org/gigisathome/programs/teen-and-adult-13/#	Special Olympics School of Strength: https://www.specialolympics.org/school-of-strength
MGH Down Syndrome Program: https://www.massgeneral.org/children/down-syndrome/patient-handouts	Gigi Fit Live Online Workouts: https://gigisplayhouse.org/gigisathome/

#### 3.7.5. Physical activity components

Increased physical activity in conjunction with a reduced energy diet and behavioral counseling to assist participants with adherence to the diet and moderate to vigorous physical activity, is an important part of current multicomponent weight loss recommendations (94). However, data on the changes in physical activity in youth with DS participating in weight management interventions are limited. General physical activity education strategies, such as providing individuals with weekly physical activity recommendations, providing education around physical activity, and encouraging self-monitoring of physical activity using physical activity trackers, have not demonstrated effectiveness in promoting change in physical activity (82, 95, 96). Conversely, educating parent/caregivers (25, 97), providing greater structure and implementing scheduled activities for exercise rather than leisure exercise (80), and participating in remote group exercise classes (98), have demonstrated some effectiveness in increasing physical activity levels of adolescents with DS. Table 4 provides some online resources for increasing physical activity specific to those with DS.

#### 3.7.6. Multidisciplinary team

Youth with DS experience nutrition and medical challenges across their lifespan, such as increased risk of comorbidities, feeding/mealtime issues, and food insecurity, which may be further complicated by lack of insurance. Multidisciplinary teams which may include the primary care clinician/pediatrician, speech language pathologist, occupational therapist, physical therapist, therapeutic recreation specialist, and dietitian, are critical for long-term-care and effective weight management. For example, a weight management program that was delivered at a children's hospital and involved sessions with a child psychologist, nurse practitioner/pediatrician, dietitian, and an occupational therapist, yielded significant reductions in BMI z-score (0.02 units/month, *p* < 0.001) in 115 youth with intellectual disabilities (88).

#### 3.7.7. Self-Monitoring of diet and physical activity

Self-monitoring of diet and physical activity has been shown to be associated with decreased weight in typically developing youth (99) and in youth with DS (100). Traditional methods of self-monitoring of dietary intake include written food diaries, and traditional methods of self-monitoring of physical activity include the use of pedometers and tracking steps. However, as stated previously, these methods may be not accurate in youth with DS. In the last decade, several technology-based tools have been developed for self-monitoring of diet and physical activity which may be more appropriate for youth with DS. In typically developing youth, technology based self-monitoring of diet and physical activity may be a more effective approach than traditional paper records (99, 101). In youth with DS, compliance with self-monitoring using technology vs. paper records has been shown to be similar (63). It is recommended that families of youth with DS self-monitor diet and physical activity at least once a week for general health, and daily when actively working to lose weight. Self-monitoring can be done in whatever method works best for each family, decreases burden of tracking, and is affordable. Table 4 presents a list of technology-based tools for self-monitoring of diet and physical activity.

#### 3.7.8. Treatment limitations

There are many known limitations for families to access and participate in comprehensive, multi- component obesity treatment. These limitations include the lack of treatment programs and clinicians with experience in pediatric obesity treatment in youth with DS. Additionally, many families struggle with transportation issues, loss of school or work time, and caregiver burn-out. However, tele-health may help to increase the reach of many treatment programs as well as overcome barriers related to transportation and time. The following recommendation provides strategies that parents may be able to implement in the home.

### 3.8. Recommendation #8. Families should work to promote healthy eating and increased physical activity at home and school

The recommendations for healthy eating and increased physical activity in youth with DS are similar to those for youth without DS. As stated above, common components of weight management in youth with DS are: providing specific recommendation for the number of servings from each food group; encouraging foods with low energy density (e.g., fruits, vegetables, and lean meats) and limiting foods with high energy density (e.g., sweet and fried foods); avoiding dietary restriction; allowing individuals to have their favorite foods in moderation; and tracking dietary intake using simple pictorial displays (25, 63, 84–86). However, key factors for successful weight management in youth with DS is family involvement and family modeling (25). Table 5 provides specific strategies for families to consider in support of their children with DS adopting health-promoting behaviors. Of note, youth with DS will have individualized needs and goals; thus, working with a registered dietitian or multidisciplinary health care team could help caregivers prioritize and further individualize health goals and health promotion strategies for their family and child.

**Table 5:** Strategies for families to support their children with down syndrome to adopt health-promoting behaviors.

Promoting Healthy Eating	Promoting Physical Activity
**At Home:**	**At Home:**
Act as role models in eating healthy foods.	Build exercise into the weekly routine, and schedule when you will do it
Avoid using food as a reward.	Consider remote physical activity programs designed for youth with DS.
Involve children in planning meals, food shopping and cooking (if able).	Consider ways to be active as a family: dance to music, take walks/hike, play outside games.
Introduce 1–2 new foods every week.	Consider involving the child in physical chores such as raking leaves or sweeping or carrying groceries as a way to promote movement in a positive way: helping!
Include at least one safe food that the child likes in every meal.	Engage in and teach the family about active living - using the surrounding streets and buildings to be active every day: walk to school and work, walk to stores and restaurants, take the stairs, get off one stop early when using public transportation and walk the rest of the way to the destination.
Avoid pressuring child to try the new foods	Get involved in Special Olympics or community-based physical activity groups.
Don't stress if the child does not eat the food right away, it can take many attempts for a child to accept a new food.	**At Childcare or School:**
Limit energy dense foods such as sweets and chips to 1–2 times a week.	Explore ways to increase physical activity during the school day., e.g., movement breaks as sensory accommodations and travel training as a way to practice independence and improve tolerance for walking.
Offer a fruit or vegetable with every meal, including snacks.	Request PT/OT (physical therapy/occupational therapy) services to address readiness to access the K-12 physical education (PE) curriculum in Early Intervention years and for preschool IEP.
Portion snacks in advance.	Recommend that a physical education teacher be included on the child's IEP team and include physical therapy and/or physical activity goals in the child's IEP that support them accessing the K-12 physical education (PE) curriculum
Offer water instead of sugar-sweetened beverages (Soda, juice, chocolate milk)	Request an assessment for adaptive physical education (APE) services if a child is not successful in the general PE program.
Use positive language when talking about food or body weight.	Be sure recess is not limited or taken away as a punishment or to provide services.
Eat meals together as a family.	
Remove screens (TVs, tablets, and phones) when eating.	
Offer the child choices at each meal (Do you want blueberries or strawberries with dinner?)	
Consider modifying the texture of foods that align with the child's texture preferences and swallowing abilities.	
**At Childcare or School:**	
Review monthly school menus and pack a lunch/snack if needed. Pack water or flavored water instead of juices for lunch/snack.	
Include healthy eating goals and alternatives to food rewards in Individual Education Plan (IEP) and Transition Plans. For example, request food not be given as a reward in school settings.	
Develop behavior plans that do not use food as a reward. Document specific alternatives to food-based rewards.	

## 4. Discussion

Youth with DS are at increased risk for obesity due to both behavioral and biologic reasons. While we await the results of additional research on the etiology of obesity and on evidence-based weight management practices in youth with DS, these recommendations can be implemented by clinicians working with youth with DS as well as the family, school, and other relevant entities. These recommendations should be revised and updated as additional evidence-based practices are developed. Table 6 provides a summary of all recommendations.

**TABLE 6:** Summary of weight management recommendations for youth with Down syndrome.

**Recommendation 1: Youth with Down syndrome should be screened routinely for overweight and obesity.**
Children with Down syndrome should be screened annually for overweight and obesity. Weight and height should be measured on a standard stadiometer and scale, ideally with the individual in a gown without shoes on.
BMI should be plotted on the Down syndrome-specific growth chart for youth up to age 10, and for children over the age of 10, BMI should be plotted on both the Down syndrome -specific growth chart and the CDC growth chart.
**Recommendation 2: Clinicians and families should be aware of health conditions and risk factors that are common in Down syndrome and may impact the development of obesity.**
Down syndrome is associated with several health conditions that have independent associations with dietary intake and physical activity pattern of youth with Down syndrome.
Clinicians working with youth with Down syndrome should screen for and monitor these health conditions to aid in the prevention or treatment of obesity, and families should be aware of how these risk factors may influence diet or physical activity
**Recommendation 3: Clinicians should screen for feeding difficulties in all youth with Down syndrome.**
Feeding difficulties are common among infants, children, and adolescents with Down syndrome.
Feeding difficulties, changes in feeding, or changes in respiratory symptoms with feeding should be reviewed during medical visits
**Recommendation 4: Clinicians should include assessments of dietary intake and physical activity at every visit.**
Collection of dietary intake and physical activity are essential for prescribing appropriate energy intake for both weight loss and maintenance, providing feedback to participants in weight management programs, describing changes over time, and determining the effectiveness of the program.
Assessment of dietary intake can be done by having a parent or caregiver record all foods and beverages consumed for 3-days prior to the medical visit using a simple diet journal or a smart phone app such as Lose it! or MyFitnessPal.
Assessment of physical activity can be done with self-reported questionnaires when a patient comes into the clinic or having the youth with DS wear a physical activity tracker.
**Recommendation 5: Clinicians and families should set appropriate recommendations for dietary intake.**
Youth with Down syndrome may have a significantly lower resting metabolic rate and total daily energy expenditure relative to body size and composition.
The estimated energy requirement equations developed for children by the National Academy of Medicine, previously the Institute of Medicine, provides the most accurate prediction of energy needs in youth with Down syndrome.
The macronutrient (i.e., fat, protein, carbohydrates) needs of youth with Down syndrome are not different than youth without DS. Thus, it is recommended to refer to the national dietary recommendations when providing dietary recommendations for youth with Down syndrome.
**Recommendation 6: Clinicians and families should set appropriate recommendations for physical activity.**
When setting goals for physical activity clinicians should consider current youth recommendations but take into consideration youths with Down syndrome current activity levels, sedentary time, cardiovascular health, and intrapersonal, parental, and environmental barriers to physical activity.
Physical activity programs should be enjoyable, accessible, structured, adapted to the needs and abilities of youth with Down syndrome, and should promote social interactions.
**Recommendation 7: Clinicians should provide multi-component behavioral weight management treatment programs specific to the needs of youth with Down syndrome and with overweight or obesity.**
The US Preventive Services Task Force Recommendation Statement on Screening for Obesity in Children and Adolescents recommends ≥ 26 contact hours over 12 months to improve weight status in typically developing children and adolescents. However, intervention programs with fewer contact hours may still be effective for weight management in youth with DS
Weight management can be provided to youth with DS using either face-to-face delivery or tele-health. However, tele-health may help to increase the reach of many treatment programs as well as overcome barriers related to transportation and time.
Interventions should be family based; however, clinicians should assess family stressors and consider the family environment before making recommendations for family-based weight management treatments.
Common components of successful diet interventions in youth with Down syndrome are:
Providing specific recommendation for the number of servings from each food group
Encouraging foods with low energy density (e.g. fruits, vegetables, and lean meats) and limiting foods with high energy density (e.g. sweet and fried foods)
Allowing individuals to have their favorite foods in moderation
Use of simple pictorial displays
Use of portion-controlled meals
Physical activity promotion efforts need to go beyond just recommending increased physical activity and should include structure exercise and increased parent involvement.
Multidisciplinary teams which may include the primary care clinician/pediatrician, speech language pathologist, occupational therapist, physical therapist, therapeutic recreation specialist, and dietitian, are critical for long-term-care and effective weight management
Families of youth with Down syndrome should self-monitor diet and physical activity at least once a week for general health, and daily when actively working to lose weight. Self-monitoring can be done in whatever method works best for each family.
**Recommendation 8: Families should work to promote healthy eating and increased physical activity at home and school.**
The recommendations for healthy eating and increased physical activity in youth with DS are similar to those for youth without DS.
Parents and caregivers should:
Act as role models in eating healthy foods.
Avoid using food as a reward.
Involve children in planning meals, food shopping, and cooking (if able).
Introduce 1–2 new foods every week and include at least one safe food the child likes in every meal.
Avoid pressuring child to try the new foods.
Limit energy dense foods such as sweets and chips to 1–2 times a week.
Offer a fruit or vegetable with every meal, including snacks.
Offer water instead of sugar-sweetened beverages (Soda, juice, chocolate milk)
Use positive language when talking about food or body weight.
Build exercise into the weekly routine
Consider remote physical activity programs designed for people with Down syndrome.
Consider ways to be active as a family: dance, take walks or hike, play outside.
Involve the child in physical chores such as raking leaves or sweeping.
Get involved in Special Olympics, team, community-based, or school sports.
Be sure to include physical education and physical activity goals in the child's IEP.

There are several limitations to be considered when reviewing these recommendations. First, most interventions have been conducting in adolescents with DS and may not be effective in younger children who are more reliant on caregivers and who have unique considerations for growth. Next, the present recommendations are for behavioral weight management, thus, other obesity treatment options, such as pharmacotherapy and bariatric surgery, were not included in our recommendations and could be considered for weight management. Finally, we did not include recommendations for specific laboratory assessments to be included during clinical screening as these have been covered in detail in the Health Supervision for Children and Adolescents with Syndrome Guidelines (34).

The current recommendations highlight that weight management interventions for youth with DS are not drastically different than those for typically developing youth, clinicians should consider the unique characteristics of youth with DS, such as the increased prevalence of DS-related health conditions that may impact diet and physical activity, feeding difficulties, difficulty assessing dietary intake and physical activity, challenges and barriers for increased physical activity, and increased reliance on parents and caregivers who often experience increased stress. While youth with DS are at increased risk for obesity, recent findings demonstrate that weight management for this population is feasible with proper screening and intervention strategies. Clinicians and families should work together to determine what strategies works best for each person and their family.
